# Clinicopathologic predictors of metastasis of different regional lymph nodes in patients intraoperatively diagnosed with stage-I non-small cell lung cancer

**DOI:** 10.1186/s12885-019-5632-2

**Published:** 2019-05-14

**Authors:** Fei Zhao, Fu-Xi Zhen, Yue Zhou, Chen-Jun Huang, Yue Yu, Jun Li, Qi-Fan Li, Cheng-Xiang Zhu, Xiao-Yu Yang, Shu-Hui You, Qian-Ge Wu, Xue-Yun Qin, Yi Liu, Liang Chen, Wei Wang

**Affiliations:** Department of Cardiothoracic Surgery, First Affiliated Hospital of Nanjing Medical University, 300 Guangzhou Road, Nanjing, 210029 China

**Keywords:** Non-small cell lung cancer, Regional lymph node, Metastasis, Multivariable logistic regression

## Abstract

**Background:**

Selection of the best lymph node for dissection is a controversial topic in clinical stage-I non-small cell lung cancer (NSCLC). Here, we sought to identify the clinicopathologic predictors of regional lymph node metastasis in patients intraoperatively diagnosed with stage-I NSCLC.

**Methods:**

A retrospective review of 595 patients intraoperatively diagnosed as stage I non-small-cell lung cancer who underwent lobectomy with complete lymph node dissection was performed. Univariate and multivariable logistic regression analysis was performed to determine the independent predictors of regional lymph node metastasis.

**Results:**

Univariate logistic regression and multivariable analysis revealed three independent predictors of the presence of metastatic hilar lymph nodes, five independent predictors for lobe specific mediastinal lymph nodes, two independent predictors for lobe nonspecific mediastinal lymph nodes and two independent predictors for skipping mediastinal lymph nodes.

**Conclusions:**

A complete mediastinal lymph node dissection may be considered for patients suspected of nerve invasion and albumin (> 43.1 g/L) or nerve and vascular invasions. Lobe-specific lymph node dissection should probably be performed for patients suspected of pulmonary membrane invasion, vascular invasion, CEA (> 2.21 ng/mL), and tumor (> 1.6 cm) in the right lower lobe or mixed lobes. Hilar lymph node dissection should probably be performed for patients suspected of having bronchial mucosa and cartilage invasion, vascular invasion, and CEA (> 2.21 ng/mL).

**Electronic supplementary material:**

The online version of this article (10.1186/s12885-019-5632-2) contains supplementary material, which is available to authorized users.

## Background

Patients diagnosed with stage-I non-small cell lung cancer (NSCLC) are most likely to be cured by surgical radical resection. Lymph node dissection is an important part of this procedure that can improve the prognosis of the patients in stage-I [1]. However, selection of the best strategy for lymph node dissection remains controversial.

In general, lymph nodes with short-axis diameters of > 1 cm as seen on CT scan are considered by the radiologists to represent metastasis when other reasons causing lymph node enlargement, such as chronic inflammation and tuberculosis, are excluded. Unfortunately, the diagnostic accuracy of CT scan for the preoperative lymph node stage is only 45–79% [2–6]. Also, 12–17% of patients pathologically diagnosed as N2 are preoperatively considered as N0. Skipping metastasis is also found in a part of these patients, as their CT scan results revealed lymph nodes with short-axis diameters of < 1 cm [4, 5, 7]. With technological developments, many methods, such as positron emission tomography, mediastinoscopy, and endoscopic ultrasound-guided fine-needle aspiration, are used to make accurate diagnosis when the surgeon ambiguously confirms preoperative lymph node metastasis. However, owing to the invasive nature of the procedure and the associated expenses, these diagnostic methods could not be routinely used for screening patients with clinical stage-I disease. Also, these procedures yield a considerable number of false-negative results and complications [8–10].

Although systematic nodal dissection can guarantee an accurate pathological nodal (pN) staging with a sufficient quantity of lymphatic tissue, the occurrence of postoperative complications will increase. For early stage patients, getting accurate patterns of lymph node dissection will decrease the postoperative complications and speed-up the patient recovery.

Regional lymph nodes attract attention for less invasive intraoperative lymph node dissection in early stage patients. We can classify the lymph nodes into four regional lymph nodes: Interlobar lymph node, Hilar lymph nodes, Lobe-specific mediastinal lymph nodes and Lobe non-specific mediastinal lymph nodes. Skipping mediastinal metastasis is defined as the metastasis of lobe nonspecific mediastinal lymph nodes, and it is confirmed pathologically by the absence of lobe specific mediastinal lymph node metastasis. The concept of lobe specific mediastinal lymph node is based on the lobe specificity of the lymphatic spread, and the characteristic lymph nodal metastasis patterns could be derived from different primary tumor locations [11, 12]. Surgeons can design an accurate strategy for lymph node dissection according to the regulation of regional lymph node metastasis.

However, each patient exhibits different clinical and pathological characteristics. Several studies have demonstrated that the incidence of lymph node metastasis differs according to individual clinical parameters and histologic components within the tumor [13–17]. This patient heterogeneity finally affects the pattern of regional lymph node metastasis in early stage lung cancer.

The goal of this study was to identify the clinicopathologic characteristics that could predict the differences in metastasis among the various regional lymph nodes and to discuss the patterns for patients intraoperatively diagnosed with stage-I NSCLC. These clinicopathologic predictors will probably provide surgeons with useful information to select the appropriate lymph node dissections, especially for early stage patients.

## Methods

### Patient selection

A total of 595 patients who consecutively underwent surgical resection for primary lung cancer at our hospital from January 2015 to December 2017 were reviewed retrospectively. The records of patients intraoperatively diagnosed with stage-I NSCLC who underwent lobectomy or segmentectomy with complete lymph node dissection as per the nomenclature were selected for this study. All patients met the criteria for stage-I NSCLC based on the new International Staging System for NSCLC (National Comprehensive Cancer Network (NCCN) Guidelines Version 3.2014: Staging Non-Small Cell Lung Cancer) [12]. We excluded patients who exhibited any one of the following conditions: 1) preoperative tumor size > 4 cm and lymph node > 1 cm at the largest diameter on CT imaging or evidence of distant metastasis; 2) preoperative chemotherapy or radiotherapy; 3) previous or coexistent tuberculosis or malignant disease; 4) complete lymph node dissection that did not meet the current standards (i.e., all lymph node stations, including right-hand stations 2–4 and 7–9 and left-hand stations 2–9); 5) synchronous lung cancers, or 8) intraoperative frozen rapid pathological results depicting tumor size > 4 cm in the largest diameter.

Patients were preoperatively assessed with chest X-ray, chest and upper abdominal CT scan, brain magnetic resonance imaging, and bone scintigraphy. CT scan was used for preoperative N-staging. The approach for primary lung cancer resection was video-assisted thoracic surgery.

Tissue specimens contained pulmonary nodules and lymph nodes. Pulmonary nodules were analyzed using rapid frozen section in the pathology department of our medical center. The remaining nodules and lymph nodes were fixed using 10% formalin, and conventional formalin-fixed, paraffin-embedded pathological tests were performed.

### Statistical analysis

The baseline patient characteristics were summarized in percent for categorical variables. The significance of associations with the outcome of lymph nodal metastases was first evaluated using a univariate logistic analysis (*P* < 0.20). These significant variables were further analyzed by multivariable analysis as independent predictors for lymph node metastasis (*P* < 0.05). Odds ratios (ORs) with 95% confidence intervals (CIs) were calculated. Statistical analyses were performed using STATA software, release 13.

## Results

### Patient characteristics

A total of 595 patients intraoperatively diagnosed with stage-I NSCLC were included in this study. Table 1 displays the patient demographics and clinical characteristics. The mean age was 60 years (range 25–87 years). Among the patients, 304 (51.1%) patients had a maximum tumor diameter of ≤1.6 cm and 284 (47.7%) had a diameter of > 1.6 cm. The tumor originated in the right upper lobe in 181 patients (30.4%), right middle lobe in 28 (4.7%), right lower lobe in 84 (14.1%), left upper lobe in 135 (22.7%), left lower lobe in 87 (14.6%), ipsilateral mixed lobes in 48 (8.1%), and bilateral mixed lobes in 26 (4.4%). Histologically, the tumors in 442 patients (74.3%) were identified as adenocarcinoma, in 62 (10.4%) as squamous cell carcinoma, in 73 (12.3%) as in situ adenocarcinoma, and in 16 (2.7%) as others. The tumor differentiation included stage-I (125 patients, 21.0%), I–II (82 patients, 13.8%), II (139 patients, 23.4%), II–III (137 patients, 23.0%), and III (61 patients, 10.3%). Pulmonary membrane invasion was present in 58 patients (9.7%), bronchial mucosa and cartilage invasion in 54 (9.1%), vascular invasion in 44 (7.4%), and nerve invasion in 13 (2.2%). The number of patients with CEA ≤ 2.21 ng/mL was 253 (42.5%) and > 2.21 ng/mL was 254 (42.7%); albumin ≤43.1 g/L was 289 (48.6%) and > 43.1 g/L was 288 (48.4%).

**Table 1:** Patient demographics and clinical characteristics (*n* = 595)

Characteristics	Value
Age (years)	
Median (Interquartile range)	60 (25–87)
≤ 60	301 (50.6%)
> 60	294 (49.4%)
Sex	
Female	304 (51.1%)
Male	291 (48.9%)
Maximum diameter of tumor	
≤ 1.6 cm	304 (51.1%)
> 1.6 cm	284 (47.7%)
Missing	7 (1.2%)
Position	
Right Upper Lobe	181 (30.4%)
Right Middle Lobe	28 (4.7%)
Right Lower Lobe	84 (14.1%)
Left Upper Lobe	135 (22.7%)
Left Lower Lobe	87 (14.6%)
Ipsilateral Mixed lobes	48 (8.1%)
Bilateral Mixed lobes	26 (4.4%)
Missing	6 (1.0%)
Pathological type	
Adenocarcinoma	442 (74.3%)
Squamous cell carcinoma	62 (10.4%)
Adenocarcinoma in situ	73 (12.3%)
Others	16 (2.7%)
adenosquamous carcinoma	4
large cell neuroendocrine lung cancer	3
metastatic colorectal cancer	1
carcinoid	5
sclerosing pneumocytoma	1
lymphlepithelioma	1
metastatic papillary thyroid cancer	1
Missing	2 (0.35%)
Tumor differentiation	
No	28 (4.7%)
I	125 (21.0%)
I-II	82 (13.8%)
II	139 (23.4%)
II-III	137 (23.0%)
III	61 (10.3%)
Missing	23 (3.9%)
Pathological morphology	
No	210 (35.3%)
lepidic	37 (6.2%)
Acinar	178 (29.9%)
Micropapillary	4 (0.7%)
Papillary	85 (14.3%)
solid	53 (8.9%)
Missing	28 (4.7%)
Pulmonary membrane invasion	
No	431 (72.4%)
Yes	58 (9.7%)
Missing	106 (17.8%)
Bronchial mucosa and cartilage invasion	
No	540 (90.8%)
Yes	54 (9.1%)
Missing	1 (0.2%)
Vascular invasion	
No	548 (92.1%)
Yes	44 (7.4%)
Missing	3 (0.5%)
Nerve invasion	
No	581 (97.6%)
Yes	13 (2.2%)
Missing	1 (0.2%)
CEA	
≤ 2.21 ng/ml	253 (42.5%)
> 2.21 ng/ml	254 (42.7%)
Missing	88 (14.8%)
Albumin	
≤ 43.1 g/l	289 (48.6%)
> 43.1 g/l	288 (48.4%)
Missing	18 (3.0%)

### Association of Individual clinicopathologic characteristics with metastasis of different regional lymph nodes

For the interlobar lymph node, univariate analysis showed that sex (OR = 1.95, 95% CI 0.88–4.30; *P* = 0.098, male patients 9.0% and female patients 4.8%), maximum diameter of the tumor (OR = 2.75, 95% CI 1.19–6.36; *P* = 0.018, > 1.6 cm 10.0%, ≤1.6 cm 3.9%), position (*P* < 0.0001, right lower lobe 26.2%, left lower lobe 9.0%, and bilateral mixed lobes 11.8% were higher than the other lobes), pulmonary membrane invasion (OR = 2.88, 95% CI 0.98–8.47; *P* = 0.055, present 12.5% and absent 4.7%), bronchial mucosa and cartilage invasion (OR = 2.81, 95% CI 1.07–7.40; *P* = 0.036, present 15.4% and absent 6.1%), vascular invasion (OR = 2.23, 95% CI 0.72–6.88; *P* = 0.164, present 13.3% and absent 6.5%), CEA (OR = 4.61, 95% CI 1.53–13.91; *P* = 0.007, > 2.21 ng/mL 9.9% and ≤ 2.21 ng/mL 2.3%), and albumin (OR = 0.27, 95% CI 0.11–0.64; *P* = 0.003, ≤43.1 g/L 11.3% and > 43.1 g/L 3.3%) were the 8 significant risk factors associated with metastasis (Table 2).

**Table 2:** Univariate and multivariate logistic regression predictors of interlobar lymph node metastasis

Independents Variables	Univariate Predictors		Multivariate Predictors	
	N Metastasis/N total	Odds Ratio (95% CI), *P*-value	B	Odds Ratio (95% CI), *P*-value
Age (years)				
≤60	19/216 (8.8%)	Reference		
> 60	10/202 (5.0%)	0.54 (0.25–1.19), 0.540		
Sex				
Female	10/207 (4.8%)	Reference		
Male	19/211 (9.0%)	1.95 (0.88–4.30), 0.098*		
Maximum diameter of tumor				
≤1.6 cm	8/205 (3.9%)	Reference		
> 1.6 cm	21/209 (10.0%)	2.75 (1.19–6.36), 0.018*		
Position		< 0.0001*		
Right Upper Lobe	3/121 (2.5%)	Reference		
Right Middle Lobe	0/22 (0.0%)	0.00 (0.00-), 0.998		
Right Lower Lobe	16/61 (26.2%)	13.99 (3.89–50.30), < 0.0001*		
Left Upper Lobe	1/93 (1.1%)	0.43 (0.04–4.18), 0.465		
Left Lower Lobe	6/67 (9.0%)	3.87 (0.94–16.01), 0.062*		
Ipsilateral Mixed lobes	1/35 (2.9%)	1.16 (0.12–11.48), 0.901		
Bilateral Mixed lobes	2/17 (11.8%)	5.24 (0.81–33.96), 0.082*		
Pathological type				
Adenocarcinoma	25/319 (7.8%)	Reference		
Others	4/98 (4.1%)	0.50 (0.17–1.48), 0.209		
Tumor differentiation				
No	0/102 (0.0%)	Reference		
Yes	29/302 (9.6%)	171,607,233.80 (0.00-), 0.996		
Pathological morphology				
No	8/140 (5.7%)	Reference		
Yes	21/262 (8.0%)	1.44 (0.62–3.34), 0.398		
Pulmonary membrane invasion				
No	14/296 (4.7%)	Reference		
Yes	5/40 (12.5%)	2.88 (0.98–8.47), 0.055*		
Bronchial mucosa and cartilage invasion				
No	23/379 (6.1%)	Reference		
Yes	6/39 (15.4%)	2.81 (1.07–7.40), 0.036*		
Vascular invasion				
No	25/387 (6.5%)	Reference		
Yes	4/30 (13.3%)	2.23 (0.72–6.88), 0.164*		
Nerve invasion				
No	28/412 (6.8%)	Reference		
Yes	1/6 (16.7%)	2.74 (0.31–24.29), 0.365		
CEA				
≤2.21 ng/ml	4/172 (2.3%)	Reference		
> 2.21 ng/ml	18/182 (9.9%)	4.61 (1.53–13.91), 0.007*		
Albumin				
≤43.1 g/l	22/195 (11.3%)	Reference		
> 43.1 g/l	7/212 (3.3%)	0.27 (0.11–0.64), 0.003*		

*Only variables with *P* value less than 0.20 were included in the multivariate analysis

In the case of hilar lymph node, univariate analysis exposed that sex (OR = 1.83, 95% CI 1.06–3.16; *P* = 0.029, male patients 16.4% and female patients 9.7%), maximum tumor diameter (OR = 5.42, 95% CI 2.68–10.97; *P* < 0.0001, > 1.6 cm 19.9% and ≤ 1.6 cm 4.4%), position (*P* = 0.122, right lower lobe 19.1%, right middle lobe 25.0%, and ipsilateral mixed lobes 21.2% were higher than the other lobes), tumor differentiation (OR = 6.36, 95% CI 1.95–20.74; *P* = 0.002, present 16.3% and absent 3.0%), pulmonary membrane invasion (OR = 3.20, 95% CI 1.58–6.47; *P* = 0.001, present 25.5% and absent 9.6%), bronchial mucosa and cartilage invasion (OR = 5.57, 95% CI 2.94–10.56; *P* < 0.0001, present 38.5% and absent 10.1%), vascular invasion (OR = 7.68, 95% CI 3.86–15.26; *P* < 0.0001, present 46.3% and absent 10.1%), nerve invasion (OR = 5.90, 95% CI 1.75–19.95; *P* = 0.004, present 45.5% and absent 12.4%), CEA (OR = 9.66, 95% CI 3.74–24.90; *P* < 0.0001, > 2.21 ng/mL 20.1% and ≤ 2.21 ng/mL 2.5%), and albumin (OR = 0.27, 95% CI 0.11–0.64; *P* = 0.003, ≤43.1 g/L 18.2% and > 43.1 g/L 7.0%) were the 10 significant risk factors associated with metastasis (Table 3).

**Table 3:** Univariate and multivariate logistic regression predictors of hilar lymph node metastasis

Independents Variables	Univariate Predictors		Multivariate Predictors	
	N Metastasis/N total	Odds Ratio (95% CI), *P*-value	B	Odds Ratio (95% CI), *P*-value
Age (years)				
≤ 60	31/243 (12.8%)	Reference		
> 60	33/245 (13.5%)	1.07 (0.63–1.80), 0.816		
Sex				
Female	23/238 (9.7%)	Reference		
Male	41/250 (16.4%)	1.83 (1.06–3.16), 0.029*		
Maximum diameter of tumor				
≤1.6 cm	10/228 (4.4%)	Reference		
> 1.6 cm	51/256 (19.9%)	5.42 (2.68–10.97), < 0.0001*		
Position		0.122*		
Right Upper Lobe	15/157 (9.6%)	Reference		
Right Middle Lobe	5/20 (25.0%)	3.16 (1.01–9.90), 0.049*		
Right Lower Lobe	13/68 (19.1%)	2.24 (1.00–5.01), 0.050*		
Left Upper Lobe	16/110 (14.5%)	1.61 (0.76–3.42), 0.213		
Left Lower Lobe	6/80 (7.5%)	0.77 (0.29–2.06), 0.600		
Ipsilateral Mixed lobes	7/33 (21.2%)	2.55 (0.95–6.86), 0.064*		
Bilateral Mixed lobes	2/16 (12.5%)	1.35 (0.28–6.53), 0.707		
Pathological type				
Adenocarcinoma	51/370 (13.8%)	Reference		
Others	13/118 (11.0%)	0.77 (0.41–1.48), 0.439		
Tumor differentiation				
No	3/101 (3.0%)	Reference		
Yes	60/368 (16.3%)	6.36 (1.95–20.74), 0.002*		
Pathological morphology				
No	21/167 (12.6%)	Reference		
Yes	42/306 (13.7%)	1.11 (0.63–1.94), 0.725		
Pulmonary membrane invasion				
No	33/342 (9.6%)	Reference		
Yes	14/55 (25.5%)	3.20 (1.58–6.47), 0.001*		
Bronchial mucosa and cartilage invasion				
No	44/436 (10.1%)	Reference		
Yes	20/52 (38.5%)	5.57 (2.94–10.56), < 0.0001*	1.13	3.11 (1.19–8.13), 0.021^#^
Vascular invasion				
No	45/445 (10.1%)	Reference		
Yes	19/41 (46.3%)	7.68 (3.86–15.26), < 0.0001*	1.09	2.98 (1.14–7.81), 0.026^#^
Nerve invasion				
No	59/477 (12.4%)	Reference		
Yes	5/11 (45.5%)	5.90 (1.75–19.95), 0.004*		
CEA				
≤2.21 ng/ml	5/197 (2.5%)	Reference		
> 2.21 ng/ml	44/219 (20.1%)	9.66 (3.74–24.90), < 0.0001*	2.14	8.49 (2.49–28.97), 0.001^#^
Albumin				
≤43.1 g/l	45/247 (18.2%)	Reference		
> 43.1 g/l	16/227 (7.0%)	0.27 (0.11–0.64), 0.003*		

*Only variables with *P* value less than 0.20 were included in the multivariate analysis. ^**#**^ *P* < 0.05 Sum might not always be in total because of missing data

For the lobe specific mediastinal lymph node, univariate analysis established that sex (OR = 1.97, 95% CI 1.08–3.59; *P* = 0.028, male patients 11.1% and female patients 6.0%), maximum tumor diameter (OR = 4.76, 95% CI 2.33–9.74; *P* < 0.0001, > 1.6 cm 14% and ≤ 1.6 cm 3.3%), position (*P* = 0.096, right lower lobe 16.7% and right middle lobe 14.3% were higher than the other lobes), tumor differentiation (OR = 9.93, 95% CI 2.38–41.37; *P* = 0.002, present 11.6% and absent 1.3%), pulmonary membrane invasion (OR = 3.95, 95% CI 1.87–8.34; *P* < 0.0001, present 21.1% and absent 6.3%), bronchial mucosa and cartilage invasion (OR = 4.84, 95% CI 2.41–9.71; *P* < 0.0001, present 25.9% and absent 6.7%), vascular invasion (OR = 7.49, 95% CI 3.68–15.26; *P* < 0.0001, present 34.1% and absent 6.5%), nerve invasion (OR = 3.75, 95% CI 0.98–14.33; *P* = 0.053, present 25.0% and absent 8.2%), CEA (OR = 6.53, 95% CI 2.69–15.82; *P* < 0.0001, > 2.21 ng/mL 13.8% and ≤ 2.21 ng/mL 2.4%), and albumin (OR = 0.46, 95% CI 0.25–0.85; *P* = 0.013, ≤43.1 g/L 11.5% and > 43.1 g/L 5.6%) were the 10 significant risk factors associated with metastasis (Table 4).

**Table 4:** Univariate and Multivariate Logistic Regression Predictors of Lobe specific Mediastinal lymph Node Metastasis

Independents Variables	Univariate Predictors		Multivariate Predictors	
	N Metastasis/N total	Odds Ratio (95% CI), *P*-value	B	Odds Ratio (95% CI), *P*-value
Age (years)				
≤60	25/298 (8.4%)	Reference		
> 60	25/291 (8.6%)	1.03 (0.58–1.83), 0.930		
Sex				
Female	18/301 (6.0%)	Reference		
Male	32/288 (11.1%)	1.97 (1.08–3.59), 0.028*		
Maximum diameter of tumor				
≤1.6 cm	10/303 (3.3%)	Reference		
> 1.6 cm	39/279 (14.0%)	4.76 (2.33–9.74), < 0.0001*	1.16	3.18 (1.15–8.78), 0.026^#^
Position		0.096*		0.019^#^
Right Upper Lobe	9/181 (5.0%)	Reference		
Right Middle Lobe	4/28 (14.3%)	3.19 (0.91–11.15), 0.070*	1.78	5.92 (0.93–37.66), 0.060
Right Lower Lobe	14/84 (16.7%)	3.82 (1.58–9.24), 0.003*	2.42	11.29 (2.50–51.04), 0.002^#^
Left Upper Lobe	10/135 (7.4%)	1.53 (0.60–3.87), 0.371	0.06	1.07 (0.22–5.07), 0.937
Left Lower Lobe	7/87 (8.0%)	1.67 (0.60–4.65), 0.324	1.21	3.35 (0.78–14.38),0.103
Ipsilateral Mixed lobes	4/48 (8.3%)	1.74 (0.51–5.91), 0.376	1.93	6.89 (1.16–40.83),0.034^#^
Bilateral Mixed lobes	2/26 (7.7%)	1.59 (0.33–7.81), 0.566	2.54	12.69 (1.65–97.51),0.015^#^
Pathological type				
Adenocarcinoma	41/440 (9.3%)	Reference		
Others	9/147 (6.1%)	0.64 (0.30–1.34), 0.233		
Tumor differentiation				
No	2/153 (1.3%)	Reference		
Yes	48/413 (11.6%)	9.93 (2.38–41.37), 0.002*		
Pathological morphology				
No	16/209 (7.7%)	Reference		
Yes	33/356 (9.3%)	1.23 (0.66–2.30), 0.511		
Pulmonary membrane invasion				
No	27/427 (6.3%)	Reference		
Yes	12/57 (21.1%)	3.95 (1.87–8.34), < 0.0001*	1.53	4.60 (1.60–13.23),0.005^#^
Bronchial mucosa and cartilage invasion				
No	36/534 (6.7%)	Reference		
Yes	14/54 (25.9%)	4.84 (2.41–9.71), < 0.0001*		
Vascular invasion				
No	35/542 (6.5%)	Reference		
Yes	15/44 (34.1%)	7.49 (3.68–15.26), < 0.0001*	1.35	3.85 (1.26–11.78), 0.018^#^
Nerve invasion				
No	47/576 (8.2%)	Reference		
Yes	3/12 (25.0%)	3.75 (0.98–14.33), 0.053*		
CEA				
≤2.21 ng/ml	6/250 (2.4%)	Reference		
> 2.21 ng/ml	35/253 (13.8%)	6.53 (2.69–15.82), < 0.0001*	1.79	6.01 (1.86–19.44), 0.003^#^
Albumin				
≤43.1 g/l	33/287 (11.5%)	Reference		
> 43.1 g/l	16/286 (5.6%)	0.46 (0.25–0.85), 0.013*		

*Only variables with *P* value less than 0.20 were included in the multivariate analysis. ^**#**^ *P* < 0.05 Sum might not always be in total because of missing data

For the lobe nonspecific mediastinal lymph node, univariate analysis indicated that tumor differentiation (OR = 2.68, 95% CI 0.79–9.11; *P* = 0.115, present 5.1% and absent 2.0%), bronchial mucosa and cartilage invasion (OR = 3.58, 95% CI 1.36–9.45; *P* = 0.010, present 11.1% and absent 3.4%), vascular invasion (OR = 7.31, 95% CI 2.93–18.21; *P* < 0.0001, present 18.2% and absent 3.0%), and nerve invasion (OR = 8.81, 95% CI 2.22–34.93; *P* = 0.002, present 25.0% and absent 3.6%) were the 4 significant risk factors associated with metastasis (Table 5).

**Table 5:** Univariate and multivariate logistic regression predictors of lobe non-specific mediastinal lymph node metastasis

Independents Variables	Univariate Predictors		Multivariate Predictors	
	N Metastasis/N total	Odds Ratio (95% CI), *P*-value	B	Odds Ratio (95% CI), *P*-value
Age (years)				
≤60	15/298 (5.0%)	Reference		
> 60	9/291 (3.1%)	0.60 (0.26–1.40), 0.238		
Sex				
Female	14/301 (4.7%)	Reference		
Male	10/288 (3.5%)	0.74 (0.32–1.69), 0.471		
Maximum diameter of tumor				
≤1.6 cm	9/303 (3.0%)	Reference		
> 1.6 cm	14/279 (5.0%)	1.73 (0.74–4.05), 0.210		
Position		0.535		
Right Upper Lobe	5/181 (2.8%)	Reference		
Right Middle Lobe	0/28 (0.0%)	0.00 (0.00,) 0.998		
Right Lower Lobe	7/84 (8.3%)	3.20 (0.99–10.40), 0.053		
Left Upper Lobe	4/135 (3.0%)	1.08 (0.28–4.08), 0.916		
Left Lower Lobe	4/87 (4.6%)	1.70 (0.44–6.48), 0.440		
Ipsilateral Mixed lobes	3/48 (6.3%)	2.35 (0.54–10.19), 0.255		
Bilateral Mixed lobes	1/26 (3.8%)	1.41 (0.16–12.55), 0.759		
Pathological type				
Adenocarcinoma	17/440 (3.9%)	Reference		
Others	7/147 (4.8%)	1.24 (0.51–3.06), 0.635		
Tumor differentiation				
No	3/153 (2.0%)	Reference		
Yes	21/413 (5.1%)	2.68 (0.79–9.11), 0.115*		
Pathological morphology				
No	8/209 (3.8%)	Reference		
Yes	16/356 (4.5%)	1.18 (0.50–2.81), 0.705		
Pulmonary membrane invasion				
No	15/427 (3.5%)	Reference		
Yes	4/57 (7.0%)	2.07 (0.66–6.48), 0.210		
Bronchial mucosa and cartilage invasion				
No	18/534 (3.4%)	Reference		
Yes	6/54 (11.1%)	3.58 (1.36–9.45), 0.010*		
Vascular invasion				
No	16/542 (3.0%)	Reference		
Yes	8/44 (18.2%)	7.31 (2.93–18.21), < 0.0001*	1.59	4.89 (1.78–13.40), 0.002^#^
Nerve invasion				
No	21/576 (3.6%)	Reference		
Yes	3/12 (25.0%)	8.81 (2.22–34.93), 0.002*	1.55	4.73 (1.05–21.35), 0.043^#^
CEA				
≤2.21 ng/ml	8/250 (3.2%)	Reference		
> 2.21 ng/ml	14/253 (5.5%)	1.77 (0.73–4.30), 0.206		
Albumin				
≤43.1 g/l	12/287 (4..2%)	Reference		
> 43.1 g/l	11/286 (3.8%)	0.92 (0.40–2.11), 0.838		

*Only variables with *P* value less than 0.20 were included in the multivariate analysis. ^**#**^ *P* < 0.05 Sum might not always be in total because of missing data

As for skipping mediastinal lymph node, univariate analysis disclosed that age (OR = 0.38, 95% CI 0.10–1.44; *P* = 0.153, ≤60 2.7% and > 60 0%), sex (OR = 0.39, 95% CI 0.10–1.47; *P* = 0.162, female patients 2.7% and male patients 1.0%), nerve invasion (OR = 12.60, 95% CI 2.41–65.93; *P* = 0.003, present 16.7% and absent 1.6%), and albumin (OR = 0.34, 95% CI 0.19–0.62; *P* < 0.0001, > 43.1 g/L 2.8% and ≤ 43.1 g/L 1.0%) were the 4 significant risk factors associated with metastasis (Table 6).

**Table 6:** Univariate and Multivariate Logistic Regression Predictors of Skipping Mediastinal lymph Node Metastasis

Independents Variables	Univariate Predictors		Multivariate Predictors	
	N Metastasis/N total	Odds Ratio (95% CI), *P*-value	B	Odds Ratio (95% CI), *P*-value
Age (years)				
≤60	8/298 (2.7%)	Reference		
> 60	3/291 (0%)	0.38 (0.10–1.44), 0.153*		
Sex				
Female	8/301 (2.7%)	Reference		
Male	3/288 (1.0%)	0.39 (0.10–1.47), 0.162*		
Maximum diameter of tumor				
≤1.6 cm	6/303 (2.0%)	Reference		
> 1.6 cm	4/279 (1.4%)	0.72 (0.20–2.58), 0.614		
Position		0.961		
Right Upper Lobe	4/181 (2.2%)	Reference		
Right Middle Lobe	0/28 (0.0%)	0.00 (0.00,), 0.998		
Right Lower Lobe	3/84 (3.6%)	1.64 (0.36–7.49), 0.524		
Left Upper Lobe	2/135 (1.5%)	0.67 (0.12–3.69), 0.641		
Left Lower Lobe	1/87 (1.1%)	0.52 (0.06–4.67), 0.555		
Ipsilateral Mixed lobes	1/48 (2.1%)	0.94 (0.10–8.62), 0.957		
Bilateral Mixed lobes	0/26 (0.0%)	0.00 (0.00,), 0.998		
Pathological type				
Adenocarcinoma	7/440 (1.6%)	Reference		
Others	4/147 (2.75)	1.73 (0.50–6.00), 0.387		
Tumor differentiation				
No	2/153 (1.3%)	Reference		
Yes	9/413 (2.2%)	1.68 (0.36–7.87), 0.509		
Pathological morphology				
No	4/209 (1.9%)	Reference		
Yes	7/356 (2.0%)	1.03 (0.30–3.55), 0.965		
Pulmonary membrane invasion				
No	8/427 (1.9%)	Reference		
Yes	2/57 (3.5%)	1.91 (0.39–9.20), 0.423		
Bronchial mucosa and cartilage invasion				
No	10/534 (1.9%)	Reference		
Yes	1/54 (1.9%)	0.99 (0.12–7.87), 0.991		
Vascular invasion				
No	10/542 (1.8%)	Reference		
Yes	1/44 (2.3%)	1.24 (0.16–9.89), 0.841		
Nerve invasion				
No	9/576 (1.6%)	Reference		
Yes	2/12 (16.7%)	12.60 (2.41–65.93), 0.003*	3.37	29.11 (3.81–222.61), 0.001^#^
CEA				
≤2.21 ng/ml	6/250 (2.4%)	Reference		
> 2.21 ng/ml	5/253 (2.0%)	0.82 (0.25–2.72), 0.746		
Albumin				
≤43.1 g/l	3/287 (1.0%)	Reference		
> 43.1 g/l	8/286 (2.8%)	0.34 (0.19–0.62), < 0.0001*	1.63	5.09 (0.99–26.20), 0.051^#^

*Only variables with *P* value less than 0.20 were included in the multivariate analysis. ^**#**^ *P* < 0.05 Sum might not always be in total because of missing data

### Multivariable analysis of clinicopathologic characteristics associated with metastasis of different regional lymph nodes

For the interlobar lymph node, multivariate analysis of the 8 risk factors obtained from univariate analysis suggested that none of them were significant predictors of interlobar lymph node metastasis (Table 2).

For the hilar lymph node, multivariate analysis of the 10 risk factors acquired from univariate analysis showed that only bronchial mucosa and cartilage invasion (absent vs. present, OR = 3.11, 95% CI 1.19–8.13; *P* = 0.021), vascular invasion (absent vs. present, OR = 2.98, 95% CI 1.14–7.81; *P* = 0.026), and CEA (≤2.21 ng/mL vs. > 2.21 ng/mL, OR = 8.49, 95% CI 2.49–28.97; *P* = 0.001) were the 3 independent predictors associated with metastasis (Table 3).

For the lobe specific mediastinal lymph node, multivariate analysis of the 10 risk factors resulting from univariate analysis indicated that only the maximum diameter of the tumor (≤1.6 cm vs. > 1.6 cm, OR = 3.18, 95% CI 1.15–8.87; P = 0.026), position (*P* = 0.019), pulmonary membrane invasion (absent vs. present, OR = 4.60, 95% CI 1.60–13.23; *P* = 0.005), vascular invasion (absent vs. present, OR = 3.85, 95% CI 1.26–11.78; *P* = 0.018), and CEA (≤2.21 ng/mL vs. > 2.21 ng/mL, OR = 6.01, 95% CI 1.86–19.44; *P* = 0.003) were the 5 independent predictors associated with metastasis (Table 4).

For the lobe nonspecific mediastinal lymph node, multivariate analysis of the 4 risk factors obtained from univariate analysis revealed that only vascular (absent vs. present, OR = 4.89, 95% CI 1.78–13.40; *P* = 0.002) and nerve invasions (absent vs. present, OR = 4.73, 95% CI 1.05–21.35; *P* = 0.043) were the 2 independent predictors associated with the presence of metastasis (Table 5).

For the skipping mediastinal lymph node, multivariate analysis of the 4 risk factors derived from univariate analysis established that only nerve invasion (absent vs. present, OR = 29.11, 95% CI 3.81–222.61; *P* = 0.001) and albumin (≤43.1 g/L vs. > 43.1 g/L, OR = 5.09, 95% CI 0.99–26.20; *P* = 0.051) were the 2 independent predictors associated with the presence of metastasis (Table 6).

## Discussion

Evaluation of regional lymph node metastasis is important for surgeons to determine the treatment and prognosis [18]. Accordingly, regional lymph node maps have been created to standardize the assessment of metastasis. In these maps, lymph nodes are labeled using a system of numerical levels and assigned names based on their anatomical location [19, 20]. The International Association for the Study of Lung Cancer (IASLC) lymph node map is employed in the eighth edition of the TNM staging system [21]. According to the sequence of the lymph node map, lung cancer cells initially spread to the ipsilateral interlobar lymph nodes, then to the hilar lymph nodes, and finally to the mediastinal lymph nodes.

The concept of lobe specific mediastinal lymph nodes is based on the lobe specificity of the lymphatic spread [22]. In literature, lobe specific MLNs have been defined as 2R, 3, and 4R for the right upper lobe; 3, 7, and 8 for the right lower lobe; 4 L, 5, and 7 for the left upper lobe; and 4 L, 7, and 8 for the left lower lobe. However, Kotoulasa et al. and Shapiro et al. proposed a simpler pattern. Right upper lobe tumors mainly metastasize to 4R, right middle lobe to 4R and 7, right lower lobe to 7, left upper lobe to 5, and left lower lobe to 7 and 9 [11, 12]. An analysis of the recent literature led to our definitions of lobe specific lymph nodes: 2, 3, and 4 for the right upper lobe; 4 and 7 for the right middle lobe; 7 and 8 for the right lower lobe; 5 and 7 for the left upper lobe; and 7, 8, and 9 for the left lower lobe.

A complete mediastinal lymph node dissection which removes all ipsilateral mediastinal lymph nodes [23], can provide more accurate pathological staging and improved clinical outcomes for some patients. This approach is considered a standard surgical treatment for patients diagnosed preoperatively with mediastinal lymph node metastases. However, complete mediastinal lymph node dissection is not considered a routine surgical treatment for patients with stage-I NSCLC because of the increased incidence of postoperative complications including increase in blood loss, median operative time, total chest-tube drainage and occurrence rate of chylothorax. The rapid pathological results would help surgeons to make decisions about which patterns should be performed; wedge resection, segmentectomy, or lobectomy. However, surgeons do not know which pattern should be chosen for lymph node dissection and we need some guidance from clinical research. Mark Shapiro et al. further demonstrated the importance of lobe specific MLN regarded as sentinel lymph nodes in mediastinal position in the surgical treatment of early stage lung cancer [12]. Each patient exhibits different clinicopathologic characteristics that determine the risk for regional lymph node metastasis in early stage lung cancer. We attempted to identify the risk factors to predict lymph node metastasis and allow surgeons to make appropriate decisions on the extent of the dissection. For some early patients, surgeon can remove regional lymph nodes such as lobe specific MLN that are most likely to contain metastases and avoid unnecessary systemic complete lymph nodes dissection in order to accelerate patients’ postoperative recovery.

First, we used univariate analysis to ascertain the associations between clinicopathologic factors and regional lymph node metastasis. The results disclosed that sex (male patients), maximum diameter of the tumor (> 1.6 cm), position (right lower lobe, left lower lobe, and bilateral mixed lobes), pulmonary membrane invasion, bronchial mucosa and cartilage invasion, vascular invasion, CEA (> 2.21 ng/mL), and albumin (≤43.1 g/L) were the 8 significant risk factors associated with the presence of metastatic interlobar lymph nodes.

Sex (male patients), maximum diameter of the tumor (> 1.6 cm), position (right lower lobe, right middle lobe, and ipsilateral mixed lobes), tumor differentiation, pulmonary membrane invasion, bronchial mucosa and cartilage invasion, vascular invasion, nerve invasion, CEA (> 2.21 ng/mL), and albumin (≤43.1 g/L) were the 10 significant risk factors associated with the presence of metastatic hilar lymph nodes.

Sex (male patients), maximum diameter of the tumor (> 1.6 cm), position (right lower lobe, and right middle lobe), tumor differentiation, pulmonary membrane invasion, bronchial mucosa and cartilage invasion, vascular invasion, nerve invasion, CEA (> 2.21 ng/mL), and albumin (≤43.1 g/L, 11.5%) were the 10 significant risk factors associated with the presence of metastatic lobe specific mediastinal lymph nodes.

Tumor differentiation, bronchial mucosa and cartilage invasion, vascular invasion, and nerve invasion were the 4 significant risk factors associated with the presence of metastatic lobe nonspecific mediastinal lymph nodes.

Age (≤60), sex (female patients), nerve invasion, and albumin (> 43.1 g/L) were the 4 significant risk factors associated with the presence of metastatic skipping mediastinal lymph nodes.

Furthermore, multivariate analysis of these factors identified using univariate analysis suggested that all the risk factors were not significant predictors of interlobar lymph node metastasis.

Only bronchial mucosa and cartilage invasion, vascular invasion, and CEA (> 2.21 ng/mL) were the three independent predictors associated with the presence of metastatic hilar lymph nodes. Therefore, when patients are suspected of having bronchial mucosa and cartilage invasion, vascular invasion, and CEA (> 2.21 ng/mL), hilar lymph node dissection should probably be performed.

Only maximum diameter of the tumor (> 1.6 cm), position (right lower lobe, ipsilateral mixed lobes, and bilateral mixed lobes), pulmonary membrane invasion, vascular invasion, and CEA (> 2.21 ng/mL) were the 5 independent predictors associated with the presence of metastatic lobe specific mediastinal lymph nodes. Hence, when patients are suspected of pulmonary membrane invasion, vascular invasion, CEA (> 2.21 ng/mL), and tumor (> 1.6 cm) in the right lower lobe or mixed lobes, lobe specific lymph node dissection should probably be performed.

Only nerve and vascular invasions were the two independent predictors associated with the presence of metastatic lobe nonspecific mediastinal lymph nodes. Hence, when patients are suspected of nerve and vascular invasions, complete mediastinal lymph node dissection should probably be performed.

Only nerve invasion and albumin (> 43.1 g/L) were the two independent predictors associated with the presence of metastatic skipping mediastinal lymph nodes. Therefore, when patients are suspected of nerve invasion and albumin (> 43.1 g/L), complete mediastinal lymph node dissection should probably be performed.

These results demonstrate the possibility of changes to lymph node metastasis when a tumor invades different tissues. In early-stage metastasis, the tumor invades bronchial mucosa and cartilage, pulmonary membranes, and vascular tissue only. During this stage, the CEA level (> 2.21 ng/mL) is likely to be an important predictor indicating that the tumor began to metastasize from the lymphatic system. Therefore, hilar and lobe-specific mediastinal lymph nodes, which are most likely to become the first metastatic stations, should be surgically removed. In later stages of lymph node metastasis, when the tumor begins to invade vascular and neural tissues, the albumin level (> 43.1 g/L) is likely to be an important predictor that indicates and promotes skip metastasis. When there is an increased possibility of broad mediastinal metastases, a complete mediastinal lymph-node dissection is required to ensure that all suspected metastatic lymph nodes are removed.

However, our study has some limitations. This study was conducted at a single institution with retrospective methods and demonstrated the necessity of further prospective study. Further prospective study with multicenter trial should be performed to comprehensively evaluate clinicopathologic predictors of metastasis of different regional lymph nodes in patients intraoperatively diagnosed with stage-I non-small cell lung cancer.

## Conclusions

After a comprehensive analysis of results concerning the different clinicopathologic factors, we conclude that complete mediastinal lymph node dissection should probably be performed for patients suspected of nerve invasion and albumin (> 43.1 g/L) or nerve and vascular invasions; lobe specific lymph node dissection should probably be performed for patients suspected of pulmonary membrane invasion, vascular invasion, CEA (> 2.21 ng/mL), and tumor (> 1.6 cm) in the right lower lobe or mixed lobes; hilar lymph node dissection should probably be performed for patients suspected of having bronchial mucosa and cartilage invasion, vascular invasion, and CEA (> 2.21 ng/mL).

## Additional file

Additional file 1: Support file containing the Age (G), cigarettes (0:negative, 1:positive), Alcohol (0:negative, 1:positive), chronic bronchitis (0:negative, 1:positive), diabetes (0:negative, 1:positive), tumor history (0:negative, 1:positive), tumor family history (0:negative, 1:positive), blood type (0:A,1:B,2:O,3:AB), Neutrophil(G), Lymphocyte(G), N/L(G), Platelet(G), Serum Albumin(G), ALP(G), Serum Globulin(G), Al/Gl ratio(G), APTT(G), PT(G), CEA(G), CYFRA211(G), NSE(G), Tumor size(G), Tumor location (0:right upper,1:right middle,2:right lower,3:left upper,4:left lower,5:Ipsilateral), Tumor location(G), Pathology (0:AdCa,1:SqCa,2:Adenocarcinoma in situ,3:other), Pathology(G),Grade (G), Pathological morphology (1:lepidic, 2:Acinar, 3:Micropapillary, 4:Papillary, 5:solid), Pathological morphology(G), Pulmonary membrane invasion (0:negative, 1:positive), Bronchial mucosa and cartilage invasion (0:negative, 1:positive), Vascular invasion (0:negative, 1:positive), Nerve invasion (0:negative, 1:positive), Lobe-specific mediastinal lymph nodes (1:metastasis 0:no metastasis),Lobe non-specific mediastinal lymph nodes (1:metastasis 0:no metastasis), skiping mediastinal lymph nodes (1:metastasis 0:no metastasis),2, 4station(1:metastasis 0:no metastasis),5, 6station(1:metastasis 0:no metastasis),7station(1:metastasis 0:no metastasis),8station(1:metastasis 0:no metastasis), 9station(1:metastasis 0:no metastasis), hilar lymph nodes (10 station)(1:metastasis 0:no metastasis), interlobe lymph nodes (11 station)(1,metastasis 0,no metastasis) described in categorical variables and Age ranges (yrs), number of cigarettes, Tumor size (cm). Neutrophil, Lymphocyte, N/L, Platelet, Serum Albumin, ALP, Serum Globulin, Al/Gl ratio, APTT, PT, CEA, CYFRA211, NSE described in continuous variables. (XLSX 173 kb)

## Abbreviations

CEA: Carcinoembryonic antigen
CI: Confidence intervals
CT: Computed tomography
IASLC: Association for the study of lung cancer
MLN: Mediastinal lymph node
NCCN: National comprehensive cancer network
NSCLC: Non-small-cell lung cancer
OR: Odds ratio
pN: pathological nodal
TNM: Tumor, Node Metastases
